# Favorable effect of enhanced recovery programs on post-discharge mortality: a French nationwide study

**DOI:** 10.1186/s13741-022-00252-5

**Published:** 2022-05-02

**Authors:** Karem Slim, Thierry Boudemaghe, Laurent Delaunay, Lucas Léger, Frédéric Bizard

**Affiliations:** 1 University Hospital Clermont-Ferrand, Place Lucie Aubrac, 63003 Clermont-Ferrand, France; 2 University Hospital Nîmes, Montpellier, France; 3 General Clinic-Vivalto Santé Annecy, Annecy, France; 4 Department of Financial Reporting & Audit, Ecole Supérieure de Commerce de Paris, Paris, France

**Keywords:** Enhanced recovery after surgery, Mortality, Colon, Orthopedics

## Abstract

**Background:**

Enhanced recovery programs (ERPs) imply early discharge but few papers have assessed the effect of ERPs on post-discharge mortality (PDM).

**Methods:**

A multicenter nationwide case control study based on administrative data was carried out between March and December 2019. Coding for every episode of care whether in the setting of ERP or not is mandatory for hospital funding (public or private). Twelve surgical specialties or procedures were included. The episodes of care coded with ERP were matched with those without ERP code for several factors such as the type of hospital (public or private), age, gender, month of discharge, and updated Charlson score. Ninety-day PDM was the main outcome.

**Results:**

Of 420,031 patients in the database, 78,119 had an ERP code. Finally, 132,600 patients with 66,300 matched pairs were considered for the study. Overall, PDM was significantly reduced after ERPs: 0.075% vs 0.138% (*p* = 0.00042). Significant one-half and two-thirds reduction in PDM was observed respectively after hip arthroplasty (odds ratio 0.48 [95% CI 0.21–0.99]) and colectomy (odds ratio 0.36 [95% CI 0.16–0.74]).

**Conclusion:**

The findings, based on a large database and a rigorous matching, strongly suggest that ERPs reduce PDM particularly after colectomy and hip arthroplasty. This is likely due to better post-operative care in ERPs.

## Background

Enhanced recovery programs (ERPs) are now well recognized as standards of care in several specialties. As ERPs develop, we are facing increasing early discharge after surgery, with events (complications or death) occurring post-discharge.

Few studies (Zhang et al. 2020, Memtsoudis et al. 2020, Esper et al. 2020) have assessed the effect of ERPs on post-operative short-term mortality. Comparisons used historical controls or were limited to in-hospital mortality, with conflicting conclusions.

This study was necessary to explore the effect of ERPs on post-discharge mortality (PDM) and to analyze this effect by specialty.

## Methods

Aiming to assess the effect of ERPs on PDM mortality, this multicenter nationwide case control study was carried out based on data obtained from 1 March 2019 to 31 December 2019 through the French prospective payment system. In French legislation related to the research involving human participants (Law 2012–300 of March 5, 2012, modified by Order 2016–800 of June 16, 2016) informed consent is not necessary since the data were anonymous and obtained through an administrative database.

Data provide episode-level information on patient hospitalization and characteristics; each patient is uniquely identified allowing for linkage between stay records. Coding for every episode of care is mandatory for hospital funding (public or private). From early 2019, all French teams using ERPs have been able to code their care as ERP and are granted a specific financial incentive to promote this mode of care. The degree of ERP implementation was not reported. Coding care as ERP was done by each participant whatever the adherence to ERP.

Twelve surgical specialties or procedures were included. The episodes of care coded with ERP were matched with those without ERP code, on a 1:1 basis for surgical procedure. The classical post-operative mortality factors such as the type of hospital (public or private), age, gender, month of discharge (in order to take into account the possible effects of seasonality), and updated Charlson score (Quan et al. 2011) were considered for matching the groups. Therefore, two contemporary groups were thus compared: ERP vs. conventional care.

Post-discharge 90-day mortality (PDM, all causes) was the main outcome. Potential impact of ERP on PDM was studied using the Kaplan-Meier estimator. Subgroup analyses were performed for each included specialty or surgical procedure. Odds ratios were computed for overall PDM and subgroups.

## Results

Overall, 420,031 patients were retrieved in the database, of whom 78,119 had an ERP code. Finally, 132,600 patients with 66 300 matched pairs were considered on the basis of aforementioned matching criteria.

**Fig. 1:**
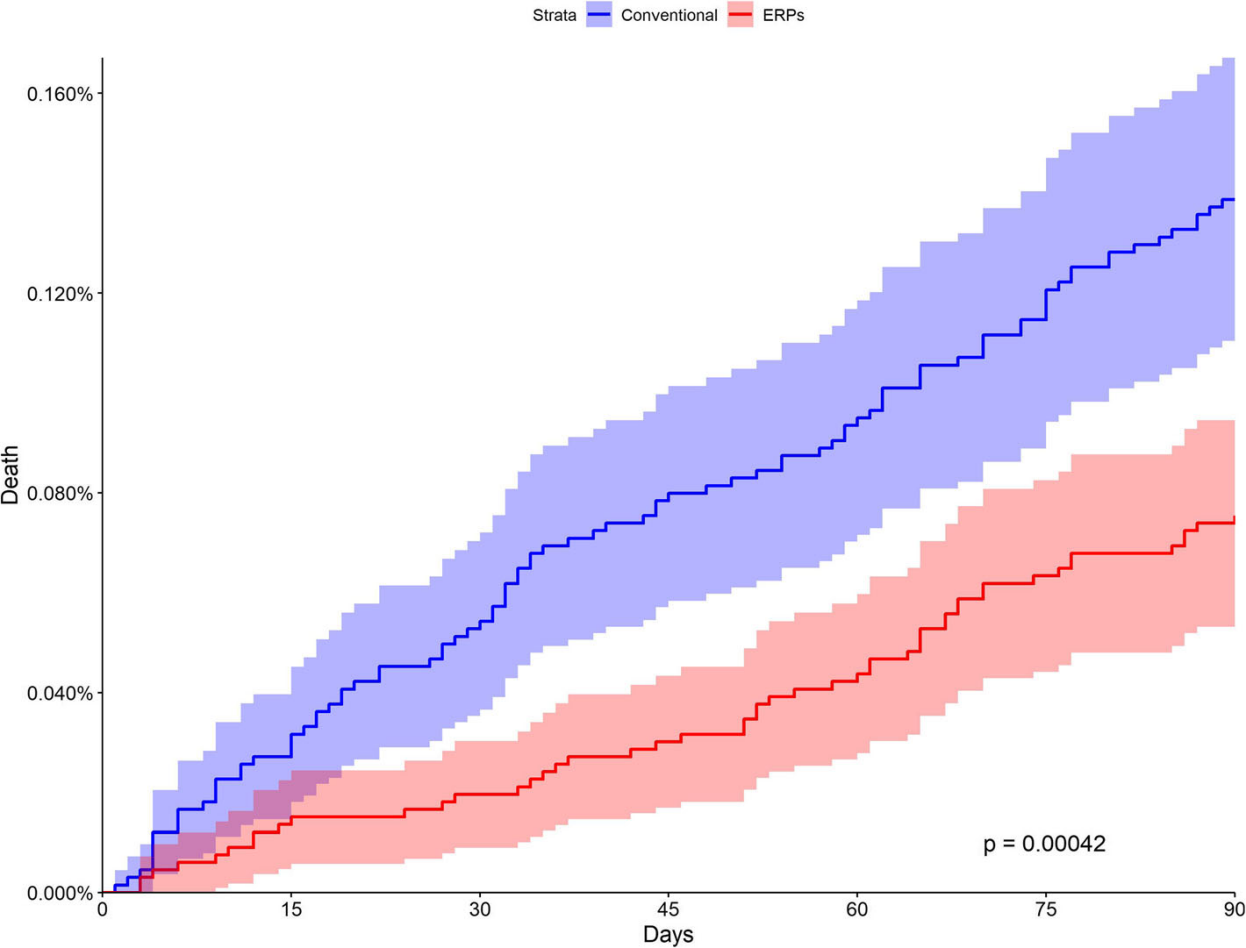
Kaplan-Meier curves of post-discharge mortality after enhanced recovery programs (red) and conventional care (blue)

Characteristics of matched groups were mean age 64.01 years, sex-ratio (male/female) 0.79, mean Charlson score 0.35, and public hospitals proportion was 29.03. Overall 90-day PDM was significantly reduced after ERPs: 0.075% vs 0.138% (*p* = 0.00042). Figure 1 shows the evolution of PDM in both groups. Table 1 shows the details of PDM rates in all included specialties or surgical procedures. Briefly, a significant one-half and two-thirds reduction in PDM was observed respectively after hip arthroplasty (odds ratio 0.48 [95% CI 0.21–0.99]) and colectomy (odds ratio 0.36 [95% CI 0.16–0.74]). The differences were not statistically significant for the other specialties.

**Table 1:** Post-discharge mortality after conventional versus ERPs by surgical specialty or procedure

Specialty or surgical procedure	Matched groups (***n*** per group)	PDM after conventional	PDM rate (%) after conventional	PDM after ERP	PDM rate (%) after ERP	Odds ratio [^**95%**^CI]	***P*** value
Hip arthroplasty	21,436	21	0.097	10	0.046	0.48 [0.21–0.99]	0.0457
Knee arthroplasty	20,726	15	0.077	11	0.053	0.73 [0.33–1.59]	0.4317
Shoulder arthroplasty	1352	2	0.148	2	0.148	1 [0.12–8.34]	1.0000
Lumbar spine surgery	5524	1	0.18	1	0.18	1 [0.04–25.29]	1.0000
Cervical spine surgery	1992	0	0	0	0	/	/
Bariatric surgery	5098	1	0.019	1	0.019	1 [0.04–25.29]	1.0000
Colectomy with anastomosis	2963	25	0.844	9	0.303	0.36 [0.16–0.74]	0.0050
Anterior rectal resection	1380	6	0.435	3	0.217	0.5 [0.11–1.9]	0.3119
Hysterectomy for benign diseases	2196	0	0	1	0.045	/	0.2390
Hysterectomy for malignancy	587	4	0.681	1	0.170	0.25 [0.11–1.9]	0.1642
Radical prostatectomy	1835	0	0	0	0	/	/
Lung resection for malignancy	1211	17	1.404	11	0.908	0.64 [0.29–1.36]	0.2523
**All**	**66,300**	**92**	**0.138**	**50**	**0.075**	**0.54 [0.38–0.76]**	**0.00042**

*Abbreviations*: *ERP* Enhanced recovery programs, *PDM* Post-discharge mortality, / = no possible calculation

## Discussion

To our knowledge, the present study is the largest one to show a significant reduction of overall PDM associated with ERPs. This finding based on a comparison of contemporary groups, refines those from historical comparisons or underpowered trials. The effect size is nevertheless more significant and relevant for hip arthroplasty and colectomy. For the other specialties, we assume that there is a lack of statistical power, larger studies are necessary given their very low PDM. A selection bias in ERP group is likely since mortality rates were low as compared with the mortality in other French studies (Manfredi et al. 2017). However, after matching for several mortality factors, our findings suggest that in low-risk patients, ERPs do reduce post-operative mortality. This observational study was not designed to address the mechanism of PDM reduction or cause of death, which will need further research. Several post-operative complications such as cardiac ischemia, delirium and cognitive dysfunction, thrombolembolic events are known to impact PDM. The well-documented reduction in post-operative complications after ERP might therefore contribute to the reported decrease in PDM. We can discuss some other causes such as better nutritional status, fewer thromboembolic events, and better post-discharge risk management. Furthermore, despite likely different degree of ERP implementation we observed a favorable effect on PDM. We think that these results are probably related to better post-discharge care owing to established clinical pathway. A recent study from Germany, in colorectal cancer field, showed the same favorable results owing to a formal inpatient rehabilitation (Scherer-Trame et al. 2021).

This study has some limitations: it is based on administrative data, but the large number of patients and rigorous matching (including hospital characteristics) offset this weakness. Since ERP coding was financially incentive, ERP was probably not under-represented. One can advocate a Hawthorne effect, but this is unlikely since the study was retrospective and based on the real life. Nevertheless, these are preliminary findings, to be confirmed in other countries.

The strengths are the size of the cohort, and that comparison is based on contemporary groups, escaping the biases of historical controls and that it is real life, enhancing its external validity.

## Conclusion

In conclusion, our finding strongly suggests that ERPs reduce PDM particularly after colectomy and hip arthroplasty. This finding is likely the results of a better post-operative care in the setting of ERPs.

## Data Availability

The datasets used and/or analyzed during the current study are available on reasonable request to K Slim or T Boudemaghe.

## Abbreviations

ERP: Enhanced recovery programs
PDM: Post-discharge mortality

## References

[CR1] Esper SA, Holder-Murray J, Subramaniam K, Boisen M, Kenkre TS, Meister K, et al. Enhanced recovery protocols reduce mortality across eight surgical specialties at Academic and University-Affiliated community hospitals. Ann Surg. 2020; 10.1097/SLA.0000000000004642. 10.1097/SLA.0000000000004642 33214486

[CR2] Manfredi S, Jooste V, Gay C, Faivre J, Drouillard A, Bouvier A-M (2017). Time trends in colorectal cancer early postoperative mortality. A French 25-year population-based study. Int J Colorectal Dis..

[CR3] Memtsoudis SG, Fiasconaro M, Soffin EM, Liu J, Wilson LA, Poeran J, Bekeris J, Kehlet H (2020). Enhanced recovery after surgery components and perioperative outcomes: a nationwide observational study. Br J Anaesth..

[CR4] Quan H, Li B, Couris CM, Fushimi K, Graham P, Hider P (2011). Updating and validating the Charlson comorbidity index and score for risk adjustment in hospital discharge abstracts using data from 6 countries. Am J Epidemiol..

[CR5] Scherer-Trame S, Jansen L, Arndt V, Chang-Claude J, Hoffmeister M, Brenner H (2021). Inpatient rehabilitation therapy among colorectal cancer patients - utilization and association with prognosis: a cohort study. Acta Oncol..

[CR6] Zhang X, Yang J, Chen X, Du L, Li K, Zhou Y (2020). Enhanced recovery after surgery on multiple clinical outcomes: Umbrella review of systematic reviews and meta-analyses. Medicine (Baltimore)..

